# Protocol: a ‘One health’ two year follow-up, mixed methods study on antibiotic resistance, focusing children under 5 and their environment in rural India

**DOI:** 10.1186/s12889-015-2632-2

**Published:** 2015-12-30

**Authors:** Cecilia Stålsby Lundborg, Vishal Diwan, Ashish Pathak, Manju R. Purohit, Harshada Shah, Megha Sharma, Vijay K. Mahadik, Ashok J. Tamhankar

**Affiliations:** Department of Public Health Sciences, Global Health - Health Systems and Policy (HSP): Medicines, focusing antibiotics, Karolinska Institutet, 171 77 Stockholm, Sweden; Department of Public Health and Environment, R.D. Gardi Medical College, Ujjain, India; International Center for Health Research, R.D. Gardi Medical College, Ujjain, India; Department of Pediatrics, R.D. Gardi Medical College, Ujjain, India; Department of Women and Children’s Health, International Maternal and Child Health Unit, Uppsala University, Uppsala, Sweden; Department of Pathology, R.D. Gardi Medical College, Ujjain, India; Central Clinical Laboratory, Ujjain Charitable Trust Hospital and Research Centre, Ujjain, India; Department of Microbiology, R.D. Gardi Medical College, Ujjain, India; Department of Pharmacology, R.D. Gardi Medical College, Ujjain, India; Ujjain Charitable Trust Hospital and Research Centre, Ujjain, India; Indian Initiative for Management of Antibiotic Resistance, Department of Environmental Medicine, R.D. Gardi Medical College, Ujjain, India

**Keywords:** One health, Health seeking behaviour, Antibiotic prescribing, Formal and informal health care providers, *Escherichia coli* in stools of children and water, Antibiotic resistance, Molecular basis of resistance

## Abstract

**Background:**

Antibiotic resistance has been referred to as ‘the greatest malice of the 21^st^ century’ and a global action plan was adopted by the World Health Assembly in 2015. There is a wealth of independent studies regarding antibiotics and resistant bacteria in humans, animals and their environment, however, integrated studies are lacking, particularly ones that simultaneously also take into consideration the health related behaviour of participants and healthcare providers. Such, ‘One health’ studies are difficult to implement, because of the complex teamwork that they entail. This paper describes the protocol of a study that investigates ‘One health’ issues regarding antibiotic use and antibiotic resistance in children and their environment in Indian villages.

**Methods/Design:**

Both quantitative and qualitative studies are planned for a cohort of children, from 6 villages, and their surrounding environment. Repeated or continues data collection is planned over 2 years for quantitative studies. Qualitative studies will be conducted once. Studies include parents’ health seeking behavior for their children (1–3 years of age at the onset), prescribing pattern of formal and informal healthcare providers, analysis of phenotypic antibiotic resistance of *Escherichia coli* from samples of stool from children and village animals, household drinking water, village source water and waste water, and investigation on molecular mechanisms governing resistance. Analysis of interrelationship of these with each other will also be done as basis for future interventions. Ethics approval has been obtained from the Institutional Ethics Committee R.D. Gardi Medical College, Ujjain, India (No: 2013/07/17-311).

**Discussion:**

The findings of the study presented in this protocol will add to our knowledge about the multi-factorial nature of causes governing antibiotic use and antibiotic resistance from a ‘One health’ perspective. Our study will be the first of its kind addressing antibiotic use and resistance issues related to children in a One-health approach, particularly for rural India.

## Background

Antibiotic resistance (ABR) is a major global public health problem, which has attracted considerable attention and research efforts in the recent past [1]. The factors associated with the global rise in ABR are multi-factorial and thus require multi-pronged strategies to prevent further development and spread of resistance [1, 2]. WHO emphasizes in the ‘Global Action Plan on Antimicrobial Resistance’ published in 2015 the need for a ‘Whole-of-society engagement including a one-health approach’ especially when it comes to antibiotic resistance [2]. The realization of the multi-factorial nature of causes governing antibiotic use and ABR has brought into focus the need for an integrated approach to study the problem in its entirety and points to the necessity to devise comprehensive interventions addressing multi-factorial issues - the ‘One health’ approach. ‘One health’ is defined as “the collaborative multi-disciplinary team-working locally, nationally, and globally — to attain optimal health for people, animals and the environment” [3]. However, as ‘One health’ is a rather new concept and as ‘One health’ studies entails an exhaustive complex teamwork, they are not common. Thus, to get a comprehensive picture of antibiotic use and resistance in a community, we need to study human behavior, (in the context of health seeking and prescribing) in their natural ecosystem including surrounding animals, source water in their geographical location, household drinking water and waste water.

All antibiotic use, especially irrational use like overuse is since long one of the major concern for development and spread of ABR in humans [4]. There have however been a limited number of studies aiming to identify context specific barriers and facilitators for appropriate clinical management of infectious diseases in lower-middle income countries like India. Identifying barriers and facilitators is a major challenge in designing and implementing successful interventions in general and for child health in particular [5]. There is need to document the current prescribing patterns along with knowledge and attitudes in relation to treatment of common infectious aetiologies by health care practitioners on one hand and also to understand the community health seeking behaviour on the other hand in order to limit the spread of ABR [6].

Apart from fundamental applications in preventing and treating infections in humans, antibiotics are used e.g. in agriculture [7]. Due to incomplete metabolism of ingested antibiotics or disposal of unused antibiotics, antibiotics enter the environment [8]. In environment, antibiotic residues might induce the development of ABR genes in the bacteria [8]. There is worldwide concern about emergence of ABR in bacteria carried by healthy individuals and in individuals in the community treated with antibiotics [9]. Commensal bacteria of the gut may play a crucial role in the spread of resistance within a community as faecal flora act as a reservoir of ABR genes [9]. Exposure of commensals such as *Escherichia coli* to antibiotics increases the carriage level of resistant bacteria by healthy humans and resistance might then also be transmitted to more virulent bacteria [9]. *E. coli* of animal, humans and environmental origin including that from water sources serve as natural habitats for ABR genes [8, 10]. As mentioned above, antibiotic residues in the environment not only alter the ecology of the environment but also give rise to selection of ABR [8]. It has also been found that the biophysical and socio-behavioral environment e.g. prescriber behavior and attitudes modifies antibiotic use and ABR [11, 12].

Since, there has been no major comprehensive study initiated in a lower-middle income country involving humans, animals, and their natural ecosystems and linking the socio-behavioral aspects of healthcare providers and health care seekers, with it, our research team initiated a trans-disciplinary ‘One health’ study with the following objectives:

- To determine, over a period of two years, patterns of health seeking behavior of parents for their children aged between 1 and 3 years in a cohort.
- Surveillance of antibiotic prescription pattern for common childhood illnesses by formal and informal health care providers (IHCPs) in the general area of the cohort of children between 1 and 3 years of age as mentioned in a).
- To explore reasons for and factors influencing antibiotic prescribing by formal and IHCPs for common childhood illnesses, including infectious diseases.
- To identify the pattern and epidemiological determinants of antibiotic resistance in *E. coli* isolated from stools of children between 1 and 3 years of age from the cohort of children as mentioned in a) and *E. coli* from drinking water in the same households and further from animals (stool) and aquatic environment (source water like wells/tube-wells/bore-wells, and waste water in included villages) over a period of two years.
- To establish the association if any, between determinants influencing health-seeking behavior of caregivers’, prescription pattern of healthcare providers and antibiotic resistance pattern of *E. coli* isolates from stools of children.
- To determine antibiotic resistance pattern over time between *E. coli* isolated; from children, household drinking water, source water, animals and waste water samples.
- To evaluate genomic commonality and diversity and pan-genomic correlates of ABR genes of *E. coli* isolated from human, animal and water samples.

## Methods

### Design and setting

#### Design

This is the protocol of a prospective cohort study using mixed methods with data collection over a two year period.

#### Setting

**Fig. 1:**
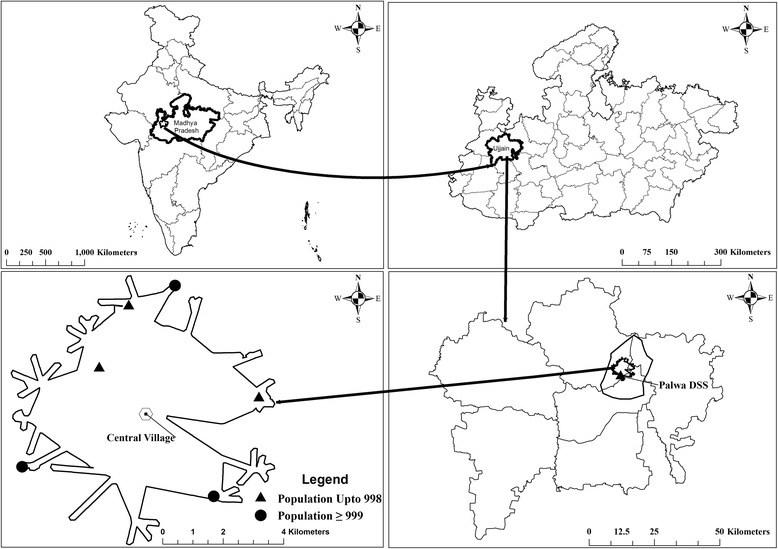
Geographical location of the six included villages. The maps show clockwise, India, Madhya Pradesh, Ujjain district and Palwa demographic surveillance site (DSS) respectively

The study will be conducted in Ujjain District in the state of Madhya Pradesh (MP), India. MP has a low Human Development Index (HDI) score of 0.45 [13]. It is the second largest state in India with almost 75 % of its population living in rural areas [14]. Specifically, the studies will be carried out in the rural Demographic Surveillance Site (DSS) of R.D. Gardi Medical College (RDGMC), Ujjain which caters to 60 villages in three development blocks (Fig. 1).

The population of these villages ranges from 800 to 2500 and approximately 50 % of the villages have a population of below 1000. Most (80 %) of the healthcare providers in the DSS (both formal and informal) are concentrated in one village, hereafter referred to as ‘central village’ and very few practice from any other village. The study area has distinct seasons viz. summer, monsoon, post monsoon and winter. The summer season (March to June) is very hot with average maximum temperatures ranging between 35 and 40 °C, while the monsoon season (late June to September) witnesses an average of 172 mm rainfall, with average maximum temperatures ranging between 29 and 31 °C. The post monsoon season/winter season (October to February) has average maximum temperatures ranging between 27 and 31 °C.

#### Selection of included villages

Since the central village had the maximum concentration of health care providers (Fig. 1) and as we are studying health seeking behavior of care givers as well as prescribing behavior of healthcare providers, we created a 5 km zone around the central village and enlisted all the villages within the zone. For further selection, villages with a population of at least 500 inhabitants and at least 15 children available in the desired age range were listed. It was also required to have a transport time of less than 45 min from each village to the central microbiology laboratory at RDGMC. Thus, six villages lying in various directions and located within 5 km from the central village were selected for the study, three villages having population > =999 and three villages having population up-to 998.

#### Study participants

To select the study participants, a baseline survey and line listing of the households was conducted in the six selected villages by trained research assistants. A three digit number code is allotted to each household in the selected villages. Members in the line-listed households are identified and their demographic characteristics (head of the household, age, sex) are recorded in the basic survey form (Table 1). Individual identification number is given to each person in the village. Then all households with children between 1 and 3 years of age with further criteria of residing in the village for the past one year, planning to continue to live in the same village for the coming three years and willing to participate in our study are listed. This list of households with children constitutes our sampling frame. Simple random sampling using random number tables is then used to select one child in the correct age per household to get the final list of children from the sampling frame. If more than one child in the specified age range is present in a household, the youngest one is included.

**Table 1:** Forms used for collection for epidemiological information in a ‘One health’ study in Ujjain, India

Form no	Form name	Sample	Description
E-00	Line listing form	Universe (*n* = 6 villages)	Enumeration of households and identification of head of the household
E-01	Basic survey form	Universe (*n* = 6 villages)	Demographic details of all the members in six villages for the identification of children for study cohort
E-02	Baseline form (SES form)	Universe (*n* = 6 villages)	Socio- economic details of all households in six villages identified during line listing
E-03	New Unique ID	Sample (*n* = 110)	Unique identification allotted children in study cohort
E-04a	Human and animal stool and water and waste water sample collection form	Sample (*n* = 110)	Forms for filling information during sample collection (Human, animal and water)
E-05	Providers prescription form	----	Forms for Surveillance of drug (including antibiotics) by health providers in study area
E-06a	Health Seeking (Follow up page)	Sample (*n* = 110)	Follow up page to record sickness found among family members of study cohort
E-06b	Health Seeking (Diary)	Sample (*n* = 110)	To record detail of health seeking by sick family members

#### Sample size calculation

Estimates of proportions from a single batch of 100 observations e.g. children (but equally applicable to prescriptions or other observations) have a precision of +/- 10%. Using repeated batches of 100 children each (every four months per year) has 80 % power to detect a linear trend in any observation in proportion of 0.1 per time unit (step between batches, negative or positive). The number of children included in the cohort will be increased to 110 to accommodate for possible attrition over time. This sample size gives a sufficiently high precision in estimates and at least a 80 % power in comparisons between data points.

#### Ethics approval and ethical considerations

Ethics approval has been obtained from the Institutional Ethics Committee R.D. Gardi Medical College, Ujjain, India (No: 2013/07/17-311).

Before any type of data collection begins, oral and written information in the local language, Hindi, is given. The aim of the study is explained to the participants or caregivers (parents/guardians), information is given that confidentiality will be guaranteed from the researchers and a consent form is signed. In the case where participants are children, parental/guardian written consent will be obtained. For interviews and focus group discussions permission will be obtained for recording. In case any child needs medical attention the child will be referred to health facilities run in collaboration with the medical college.

#### Pilot study

A pilot study was conducted to field test the data collection instruments and procedures and to train the research assistants. Minor changes in the data collection procedures were done after the pilot study.

### Data collection methods and laboratory methods

#### Household survey

A standardized household questionnaire, called Baseline form (Table 1) is used to survey all the households in the selected six villages (total 1093 households). The questionnaire enables the gathering of socio-economic background information of each household. This background information will be linked with information obtained from the other sub-studies.

#### Health Seeking Behaviour by caregivers and prescribing behaviour of healthcare providers

These sub-studies will focus on determining the patterns of health seeking behaviour (HSB) in the community, developing an understanding of interrelationship by analysing associations between factors that influence the HSB of caregivers for their children and ABR and the antibiotic prescribing practices of providers including formal as well as informal health care providers (IHCPs) in a rural community. GIS mapping of the households and formal and informal HCP will be done. GIS networks of health seeking routes will be mapped and analyzed.

##### Health seeking behavior for childhood illnesses

Quantitative survey: A questionnaire to be delivered in interview form is developed to record: the history of illness, whether or not any sort of treatment was given to the child, the child’s routes through the health care system and the reason for that route. The health seeking behaviour for the children will be followed every alternate day for two years by a team of study assistants along with trained health care workers. Drug use focusing on antibiotic use for infectious diseases will be an additional focus for this survey.

Qualitative methods: Qualitative exploratory interviews and Focus Group Discussions (FGD) will be done to ascertain the parent’s or caregiver’s perception of health seeking for childhood illness and antibiotic use in children between 1 and 3 years of age. The focus is to explore the knowledge, understanding, and perceptions of primary caregivers and their influence in decision making with respect to treatment seeking for common childhood illnesses and antibiotic use. A purposive sample of 15–20 caregivers of children between 1 to 3 years of age, with a variation in background factors like age, educational level and experience in caring for children, will be selected. The interviews will be conducted to explore the caregivers’ experiences during an episode of childhood illness and the reasons behind their actions, the problems they face, their beliefs, their perceptions about common childhood illnesses and antibiotic use and their opinions for improvement. Further, FGDs will be conducted with at least six groups of caregivers with various background characteristics, see above, within the study setting [15]. In each FGD 6–8 participants will be included. The FGDs will focus on exploring variations in ways how the caregivers respond to common childhood problems and how they see various options for health seeking. A guideline (with prompts) will be developed to follow the process; relevant probing will be used and all questions will be open ended [15]. Facilitators will conduct the interviews and FGDs in Hindi. The recordings from both the individual interviews and the FGDs will be transcribed by study assistants in Hindi.

##### Health care provider’s prescriptions for common childhood illnesses

Quantitative survey: Surveillance of antibiotic prescriptions by formal and informal HCPs (IHCPs) will be done. Data will be collected monthly for one year by giving the HCPs a specially designed prescription pad to collect their prescriptions in duplicate. The HCPs will be asked to write in the prescriptions in the specially designed prescription pad while prescribing to outpatients on their routine outpatient days. Prescriptions will be collected for the first two weeks or for the first 100 prescriptions every month. The prescription pad will have 200 leaflets. Thus, each pad will have capacity to collect 100 prescriptions per month in duplicate, one for the HCP and one for the research assistant. The prescription pads will be replaced with new prescription pads at the end of each month. Qualitative methods: To understand the reasons of the prescribing pattern especially of antibiotics for common illnesses in children, individual interviews will be conducted with 15–20 formal and informal HCPs. Open-ended questions will be used to explore perceptions about antibiotic use, resistance, its consequences and the factors driving this action; 2) FGDs will be conducted separately with different types of HCPs. The FGDs will be done by first presenting different case scenarios of commonly encountered medical conditions in children, and rating the individual responses as correct or incorrect on the basis of whether or not the antibiotic prescription given in the scenario was deemed necessary and then the FGD participant’s perception of giving this prescription in that particular scenario will be explored by starting a discussion using a topic guide. Six to eight participants will be included in each FGD and at least 6 FGD will be conducted [15]. Participants for the FGD will be selected considering gender variation and type of practitioner i.e. allopathic providers and IHCP. We will try to obtain knowledge of the different practitioner’s natural meetings within the district and find key-persons to help with recruitment. We will try to obtain homogeneity with respect to type of practitioner (i.e. allopathic provider or IHCP) in the FGDs to avoid missing out important data due to a hierarchical social system between different types of health care practitioners.

#### Sample collection for microbiological analyses

**Fig. 2:**
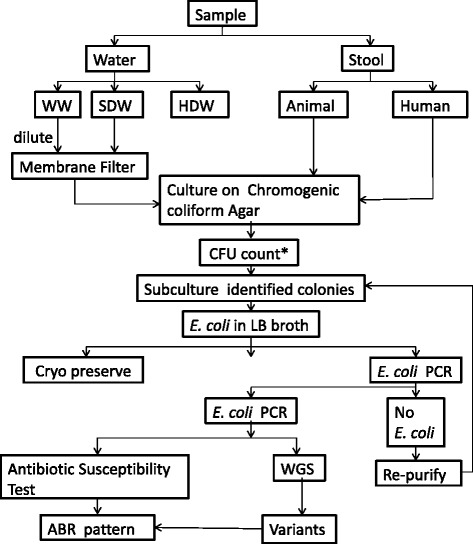
Overview of sample types included, and laboratory methods, that will be used. WW: Waste water, SDW: Source drinking water, HDW: Household drinking water, CFU: Colony forming units, *E. coli*: *Escherichia coli*, LB-broth: Luria-Bertani broth, PCR: Polymerase Chain Reaction, ABR: Antibiotic resistance, WGS: Whole Genome Sequencing

Samples will be collected at six time points during a 2-year period at an interval of four months, three times in a year in the summer, monsoon and postmonsoon/winter seasons in order to record changes over time and potential seasonal effects. An overview of samples included and laboratory methods are shown in Fig. 2.

#### Collection of stool samples from children and animals

The collection of stool samples from children and animals will be performed in the following way and with the material as described below:

*Children:* a) A stool sampling kit that includes: 1) Sterile wide mouth plastic container along with a spoon for transferring the stool to the container; 2) A sterile polyethylene sheet measuring 15x15cm for collecting stool. The container and polythene sheet to be enclosed in a zip locked polyethylene bag. b) The stool sampling kit will be distributed in the households of the selected children one day prior to the day of sample collection, c) The caregivers will be trained by the research assistants how to use the stool sampling kit to collect early morning stool on the day of sample collection, d) The parents/caregivers will be re-briefed regarding stool collection procedure at every time point of sample collection.

*Animals:* Fresh stool samples from animals will be collected in a sterile container using spatula with a precaution to avoid contamination by soil.

#### Collection of drinking water and wastewater samples

The drinking water, source water and wastewater samples will be collected in sterilized glass bottles of 1 litre capacity. Each household will be asked to give drinking water from the vessel where drinking water is stored in the house. Source drinking water will be collected from two common sources of each village and wastewater will be collected from two discharge sites in each village.

#### Transport of stool and water samples to the laboratory

Collected samples (stool and water) will be transported to the microbiology laboratory by the research assistant on the day of collection. Cold chain will be maintained to keep a temperature of 4–6 °C. The maximum time for transportation will be 45 min.

#### Laboratory methods

- *Microbiological and molecular methods*
- Isolation and confirmation of *E. coli*

*E.coli* will be isolated from the following sources: stools from cohort of children, animal stool samples, household- and source-drinking water from village and village waste- water.

The stool samples (human and animal) will be directly plated on selective (for all coliforms, inhibits other bacteria) and differential (differentiate *E. coli* from other coliforms) media HiCrome® Agar (HiMedia laboratories, Mumbai, India).

The household- and source-drinking water will be directly subjected to Membrane Filtration Technique (MFT) while waste-water will undergo sufficient serial dilution with normal saline (0.9 %) depending on the visual turbidity [16]. The filtration will be done by using through 47 mm diameter and 0.45 μm pore size nylon membrane filters for a minimum of two and half hours [16, 17]. If less than six *E.coli* colonies after 24 h of incubation, the original sample will further be filtered once with lower dilution. Following filtration the membrane will be placed on HiCrome® Coliform Agar plates incubated at 37 °C for 24 h [17]. The number of colonies of coliforms and *E.coli* (identified as blue-violet colonies on HiCrome® Coliform Agar) will be estimated after considering the dilution factor of total number of colony forming units (CFU) of coliforms and *E. coli* per 100 ml of filtered sample. The number of CFUs per 100 ml of filtered sample will be estimated for the number of total coliforms. The number of *E. coli* (identified as blue-violet colonies on HiCrome® Coliform Agar) will be counted. The comparison of CFUs will provide a snapshot of coliform load and the relative abundance of other coliforms over *E. coli* or vice versa in the samples tested [17].

All the cultured plates will be photographed. A total of six isolated colonies of *E.coli* (to get at least five confirmed *E.coli* per sample to be able to detect variation) and two colonies of different coliforms (to store for future study) will be placed in 3 ml of Luria-Bertani (LB) media [18]. The LB broth will be incubated for 24 h in shaking incubator to get sufficient growth. Thereafter 2 ml of the broth will be processed for DNA extraction while 1 ml of broth will be cryo-preserved at -80 °C in 1:1 solution with sterile autoclaved glycerol [18]. Confirmation of all *E.coli* isolates will be done by PCR (see below).

b) Antibiotic susceptibility testing

Antibiotic susceptibility testing (AST) will be performed by the Kirby-Bauer disc diffusion method. Disc strengths used will be as recommended by the current Clinical and Laboratory Standards Institute (CLSI) guidelines [19]. Antibiotics tested will be: ampicillin, ceftriaxone, cefepime, ciprofloxacin, tetracycline, tigecycline, meropenem, imipenem, gentamicin, amikacin, sulphamethoxazole, co-trimoxazole, nalidixic acid and nitrofurantoin. The choice of antibiotics is based on the CLSI guidelines and use of antibiotics in the study area for the treatment of infections caused by gram negative coliforms. The diameter of inhibition zones will be measured to the nearest millimeter independently by two technical experts. If there will be variation of more than three millimeters between two readings, the procedure will be repeated. If any further discrepancy will be there in zone diameter the susceptibility pattern will be confirmed by minimum inhibitory concentration method according to CLSI guidelines. For calculations, all isolates with intermediate susceptibility readings will be considered resistant. Results will be recorded in the form of mono-resistance (resistance to a single antibiotic), co-resistance (resistance to two antibiotics of same or different groups) and multi-drug resistance (MDR) (resistance to at least three different antibiotic classes) [20]. Extended Spectrum Beta-Lactamase (ESBL) will be detected phenotypically by the combined disc diffusion method using Muller Hinton agar with cefotaxime (30 μg) and cefotaxime/clavulanic acid (30/10 μg) and ceftazidime (30 μg) and ceftazidime/clavulanic acid (30/10 μg) according to CLSI guidelines [19].

c) Quality control

Various quality control measures will be taken at each step of the procedure to ensure laboratory quality check. The quality controls will be: a) checking of samples from each batch of all types of prepared media b) confirmation of sterility of inoculation loops, swabs and micro tips; c) check of reagents - normal saline and distilled water; d) checking of laminar flow at the beginning and at the end of procedures; Reference strains will be; *E. coli* ATCC 25922 strain for AST; an ESBL-producing *K. pneumoniae* ATCC 700603 strain as per CLSI guideline [19].

- Molecular methods
- Approach

The confirmation of all *E.coli* isolates will be done through PCR based on genus-specific primers and whole genome re-sequencing will be used for pan-genomic identification of molecular determinants of observed antibiotic resistance in *E. coli.*

b) Procedure

The genomic DNA will be extracted using standard procedures from pure *E.coli* isolates and confirmed by PCR based on genus-specific primers. The confirmed *E.coli* isolates per time point will be classified into six phenotypically different categories based on the susceptibility patterns to the tested antibiotics – purely susceptible (where the isolate is susceptible to all the fourteen antibiotics tested), purely resistant (where the isolate is resistant to all the fourteen antibiotics tested), purely intermediate (where the isolate is intermediately susceptible/resistant to all the fourteen antibiotics tested), any resistance (where the isolate is resistant to one or more but not all the fourteen antibiotics tested), any intermediate (where the isolate is intermediately susceptible/resistant to one or more but not all the fourteen antibiotics tested) and mixed (where the isolate shows a mixed combination susceptible, resistant and intermediately susceptible/resistant to the fourteen antibiotics tested). The genomic and plasmid DNA will be extracted and purified using standard procedures from confirmed and pure *E. coli* isolates which exhibit antibiotic resistance and susceptibility, respectively, to the selected antibiotics. DNA samples from all resistant isolates and from 20 % of susceptible isolates (as controls) will be subjected to whole genome sequencing using Ion-proton Technology [21]. The sequences will be assembled on the reference genome of *E. coli* available and the abundance of known antibiotic resistance genes will be estimated using the depth of coverage of the sequences assembled on the known loci of the resistance genes identified in the reference genome.

c) Quality control

PCR-positive and PCR-negative controls will be processed in all PCR assays as: a) *E. coli* ATCC 25922 strain DNA, b) positive PCR product from previous PCR assay, c) an extraction control (with all the steps but without any *E. coli*), (b) a reaction tube with substitution of distilled water for the test template, and (c) a sample that previously yielded a negative result on PCR. In a run, the PCR-negative controls will be processed first, followed by test samples and then PCR-positive control. The run will be accepted when all the PCR controls will be consistent with the results.

### Data management and analyses

#### Data management

Appropriate data software will be prepared according to the study design and outcome variables. Database software will be specially designed to ease the data entry and to link data from all sub-studies. Unique identifiers and bar codes will be used for sample tracing from the community to the laboratory and also to determine the sample flow within the laboratory. A team of senior researcher will supervise data entry. Ten per cent of data entry will be double checked by senior researchers for quality assurance. Special arrangements will be made to maintain confidentiality of the personal data. For qualitative studies FGDs and interviews, the recorded material will be stored in a locked location. Any identity of the interviewee will be removed from the transcripts before analysis. The data captured from various sub studies will be transferred to appropriate data analysis software.

#### Data analysis

All quantitative data will be analysed using STATA (StataCorp. College Station, TX, USA) and appropriate spatial GIS software.

##### Data analysis for quantitative epidemiological data

The descriptive analysis of the information provided by the care givers will provide the perceived distribution of the disease profile of the area, the patterns of health service utilisation, the factors affecting the treatment-seeking pathways including GIS pathways, the distribution of the care-seeking time (distribution number of days from the onset of symptoms to the time when the patient sought treatment from a HCP), the distribution of the healthcare expenses on the basis of illness, number of antibiotic treatments by members of the cohort and sampling frame for the formal and informal HCPs practicing in the area. Comparisons will be performed using chi-square tests/Student’s *t*-test depending on the variable type. Furthermore, we will study the differences in the outcome variables using appropriate statistical methods, like regression models and time series analyses. When relevant, clustering will be taken into account in the analyses.

##### Data analysis for quantitative laboratory data

The colony count, PCR results and antibiotic susceptibility pattern will be analysed and compared for coliform diversity and resistance pattern over a period of two years in: (a) all the stool samples from the cohort of children particularly with reference to the antibiotic use and differential proportion of antibiotic susceptibility, (b) household drinking- water of cohort of children, common drinking water source, and community waste-water, (c) stool samples from selected livestock population in-and around- the household of the selected children.

The generated genotypic data will also be analyzed (a) to identify the genotypic antibiotic resistance pattern in the *E. coli* with reference to chromosomal- and plasmid-mediated resistance using specific markers of plasmid-mediated resistance in *E. coli*, (b) to identify and confirm of the origin of *E. coli* isolates in terms of human- or animal-origin present in the gut of children (using a species-specific yet undecided primer set/gene target), (c) to identify and correlate the phylogenetic lineage of the ABR *E. coli* isolates and group them according to the identified phylogenetic groups. This will identify the predominantly circulating strains of specific phylogenetic clan in the study setting, (d) to follow, compare, and correlate the coliform diversity pattern, resistance pattern, and genetic pattern in all the human and environmental samples.

##### Combined quantitative analyses

Finally all the quantitative data will be linked together to get the overall ‘One health’ model where associations between illness patterns, health seeking behavior, prescribing and resistance from different sources (humans, animal and water) will be linked together and followed over time.

##### Data analysis for qualitative data

Appropriate data analysis method will be used for ordering and synthesising data [15, 22]. Manifest and latent content analyses will be used for the analyses [22].

## Discussion

To our knowledge this is a unique study protocol in that it plans to study the diverse and complex problem of antibiotic use and resistance utilizing both quantitative and qualitative research methods using a ‘One health’ approach [3]. The ‘One health’ approach has gained importance in public health in recent times. However, it has so far contributed very little in solving the major global health problems [23], as one of the greatest challenges faced in ‘One health’ studies is that the implementation of the studies requires collaborative efforts from experts of multiple disciplines working together in a trans-disciplinary way.

One strength of this protocol is that the research team has diverse background expertise ranging from public health, epidemiology, pharmaco-epidemiology, pharmacology, medicine, pediatrics, infectious disease, environmental medicine, agriculture, limnology, general and molecular microbiology and many of the researchers are familiar with both qualitative and quantitative methodology. The research group also has extensive experience of working together as evident from previous publications presented below.

We have earlier reported high proportion of antibiotic use for common infections among outpatients [24] and among admitted patients with a suspected focus of infections [25, 26]. High rate of antibiotic prescribing has been noted in surgical ward [25, 27], in peri-partum period [28] and in patients admitted in medical intensive care unit [29]. Inappropriate choice of antibiotics and also inappropriately long duration of antibiotic therapy for post-surgery prophylaxis has been documented [25].

High proportion of antibiotic resistance was documented in pathogens isolated from patients with suspected bacterial infections [30]. Further, resistant *E. coli* were isolated from hospital wastewater along with antibiotic residues [31–33]. High proportion of ABR was also found in commensal *E. coli* among asymptomatic pregnant women [34], post-menopausal women [35] and among healthy children [36]. ESBL-encoding genes, blaCTX-M-15 and blaTEM-1, and plasmid-mediated quinolone resistance genes, aac (6′)-Ib-cr, qnrA, qnrB and qepA, were detected form both pregnant women and healthy children [34, 36]. Our research group has also earlier demonstrated high ABR in *E. coli* isolated from cow’s stool in two geographically distinct rural areas [37, 38].

It was further found in one of our qualitative studies that people perceive that change in climate and climatic factors are associated with antibiotic resistance [39]. Presence of ABR bacteria was also detected in drinking water sources of rural areas and it was speculated that this could have been due to surface water runoff from human and/or animal fecal sources [37]. A nascent idea of the ‘One health’ approach was proposed in 2008 by one of our team members calling it ‘Location Specific Integrated Antibiotic Resistance management Strategy- LIARMS [40], and in another instance formulating it as ‘Augmented antibiotic stewardship programme’ [41].

We have earlier addressed that that behavior change in relation to antibiotics is complex and requires multi-faceted interventions [12]. The planned studies will link together resistance in *E. coli* from humans, animals and the environment in the same villages with behavior related to health-seeking and prescribing and thus create further evidence for inter-related interventions taking the ‘One-health’ concept in the field of antibiotic use and resistance into consideration.

For all the study components, the protocol employs the most appropriate design, based on the research question. Most of the community based quantitative studies are set up in a cohort of more than 100 children, a number that satisfies the statistical requirements. This cohort will be followed continuously for health-seeking behaviours and samples of analyses of *E.coli* from children and their environment (animals and various types of water) will be taken every four months for a period of two years. To our knowledge this is the first ‘One-health’ study from India simultaneously studying *E.coli* in humans, animals and the environment in the same location.

The planned molecular studies include whole genome sequencing (WGS) that is expected to provide a differential depth of coverage of genetic map for various known antibiotic resistance genes in the isolates from humans, animals and environment. This, in turn, will identify potential lead candidate genes to track in the subsequent samples from different time points. Also, such a comparative mapping between pure susceptible strains and resistant strains may identify novel genetic changes (SNPs/indels/genes/copy number variations), which could later be attributed to antibiotic resistance based on bioinformatics prediction of gene function of hitherto unidentified/unannotated DNA sequences present in the resistant genomes. The information obtained from molecular and genetic analyses will be used to correlate the possibility of sharing antibiotic resistance gene pools between *E. coli* and non-*E. coli* coliforms sharing the same macro- or micro-environment.

## Abbreviations

AB: antibiotic
ABR: antibiotic resistance
AST: antibiotic susceptibility testing
ATCC: American type culture collection
AYUSH: Ayurveda, Yoga and Naturopathy, Unani, Siddha and Homoeopathy
CFU: colony forming units
CLSI: Clinical and Laboratory Standards Institute
DNA: deoxyribonucleic acid
DSS: demographic surveillance site
*E. coli*: *Escherichia coli*
ESBL: Extended Spectrum Beta-Lactamase
FGD: Focus Group Discussion
GIS: Geographical Information System
HCP: Health Care Provider
HDI: human development index
HDW: household drinking water
HSB: health seeking behaviour
IHCP: Informal Health Care Provider
*K. pneumonia*: *Klebsiella pneumonae*
Km: kilometer
LB-broth: Luria-Bertani broth
MDR: multi-drug resistance/-t
MFT: membrane filtration technique
MP: Madhya Pradesh
PCR: polymerase chain reaction
RDGMC: R.D. Gardi Medical College
SDW: source drinking water
SNPs: single nucleotide polymorphisms
WGS: whole genome sequencing
WHO: the World Health Organization
WW: waste water

## References

[1] Michael CA, Dominey-Howes D, Labbate M (2014). The antimicrobial resistance crisis: causes, consequences, and management. Front Public Health.

[2] World Health Organization. Antimicrobial resistance Draft global action plan on antimicrobial resistance. Available at http://apps.who.int/gb/ebwha/pdf_files/WHA68/A68_20-en.pdf?ua=1. Accessed 23 Dec 2015.

[3] Min B, Allen-Scott LK, Buntain B (2013). Transdisciplinary research for complex One Health issues: a scoping review of key concepts. Prev Vet Med.

[4] World Health Organisation Improving the containment of antimicrobial resistance WHA; 2005. [http://www.searo.who.int/entity/medicines/topics/wha_58_27.pdf]. Accessed 23 Dec 2015.

[5] Lassi ZS, Mallick D, Das JK, Mal L, Salam RA, Bhutta ZA (2014). Essential interventions for child health. Reprod Health.

[6] Bebell LM, Muiru AN (2014). Antibiotic use and emerging resistance: how can resource-limited countries turn the tide?. Global Heart.

[7] Gilchrist MJ, Greko C, Wallinga DB, Beran GW, Riley DG, Thorne PS (2007). The potential role of concentrated animal feeding operations in infectious disease epidemics and antibiotic resistance. Environ Health Perspect.

[8] Economou V, Gousia P (2015). Agriculture and food animals as a source of antimicrobial-resistant bacteria. Infect Drug Resist.

[9] Macfarlane S (2014). Antibiotic treatments and microbes in the gut. Environ Microbiol.

[10] Zhang SH, Lv X, Han B, Gu X, Wang PF, Wang C, et al. Prevalence of antibiotic resistance genes in antibiotic-resistant Escherichia coli isolates in surface water of Taihu Lake Basin, China. Environ Sci Pollut Res Int. 2015. 10.1007/s11356-015-4371-4 25813640

[11] Sahoo KC. Antibiotic resistance and environmental factors : focusing on the situation in Odisha, India. Karolinska Institutet; 2012.

[12] Stålsby Lundborg C, Tamhankar AJ (2014). Understanding and changing human behaviour--antibiotic mainstreaming as an approach to facilitate modification of provider and consumer behaviour. Ups J Med Sci.

[13] Suryanarayana MH, Agrawal A, Prabhu KS. Inequality- adjusted human development index for India’s States- UNDP 2011. New Delhi. http://www.undp.org/content/dam/india/docs/inequality_adjusted_human_development_index_for_indias_state1.pdf. Accessed 23 Dec 2015.

[14] State profile Madhya Pradesh. http://www.census2011.co.in/census/state/madhya+pradesh.html. Accessed 23 Dec 2015.

[15] Malterud K (2001). Qualitative research: standards, challenges, and guidelines. Lancet.

[16] Hope MC, Neill AH (1956). The use of the membrane filter technique for testing water supplies in the field. Public Health Rep.

[17] Rice EW, Baird RB, Eaton AD, Clesceri LC. Standard methods for the examination of water and wastewater, 22nd edn. American Public Health Association, American Water Works Association, Water Environment Federation; 2012.

[18] Hollander DH, Nell EE (1954). Improved preservation of Treponema pallidum and other bacteria by freezing with glycerol. Appl Microbiol.

[19] Clinical and Laboratory Standards Institute (CLSI). Performance standard for antimicrobial susceptibility testing; twenty- third informational supplement. CLSI document M100-S23. Wayne, PA: Volume 33 No.1. In*.* 2014; p. 50-53

[20] Magiorakos AP, Srinivasan A, Carey RB, Carmeli Y, Falagas ME, Giske CG (2012). Multidrug-resistant, extensively drug-resistant and pandrug-resistant bacteria: an international expert proposal for interim standard definitions for acquired resistance. Clin Microbiol Infect.

[21] Rhodes J, Beale MA, Fisher MC (2014). Illuminating choices for library prep: a comparison of library preparation methods for whole genome sequencing of Cryptococcus neoformans using Illumina HiSeq. PLoS One.

[22] Graneheim UH, Lundman B (2004). Qualitative content analysis in nursing research: concepts, procedures and measures to achieve trustworthiness. Nurse Educ Today.

[23] Stephen C, Karesh WB (2014). Is One Health delivering results? Introduction. Rev Sci Tech.

[24] Pathak A, Mahadik K, Dhaneria SP, Sharma A, Eriksson B, Lundborg CS (2011). Antibiotic prescribing in outpatients: Hospital and seasonal variations in Ujjain, India. Scand J Infect Dis.

[25] Pathak A, Mahadik K, Dhaneria SP, Sharma A, Eriksson B, Lundborg CS (2012). Surveillance of antibiotic consumption using the “focus of infection” approach in 2 hospitals in Ujjain, India. PLoS One.

[26] Sharma M, Eriksson B, Marrone G, Dhaneria S, Lundborg CS (2012). Antibiotic prescribing in two private sector hospitals; one teaching and one non-teaching: a cross-sectional study in Ujjain, India. BMC Infect Dis.

[27] Pathak A, Saliba EA, Sharma S, Mahadik VK, Shah H, Lundborg CS (2014). Incidence and factors associated with surgical site infections in a teaching hospital in Ujjain, India. Am J Infect Control.

[28] Sharma M, Sanneving L, Mahadik K, Santacatterina M, Dhaneria S, Stalsby Lundborg C (2013). Antibiotic prescribing in women during and after delivery in a non-teaching, tertiary care hospital in Ujjain, India: a prospective cross-sectional study. J Pharm Policy Prac.

[29] Sharma M, Damlin AL, Sharma A, Stalsby Lundborg C (2015). Antibiotic prescribing in medical intensive care units--a comparison between two private sector hospitals in Central India. Infect Dis.

[30] Pathak A, Marothi Y, Kekre V, Mahadik K, Macaden R, Lundborg CS (2012). High prevalence of extended-spectrum beta-lactamase-producing pathogens: results of a surveillance study in two hospitals in Ujjain, India. Infect Drug Resist.

[31] Chandran SP, Diwan V, Tamhankar AJ, Joseph BV, Rosales-Klintz S, Mundayoor S (2014). Detection of carbapenem resistance genes and cephalosporin, and quinolone resistance genes along with oqxAB gene in Escherichia coli in hospital wastewater: a matter of concern. J Appl Microbiol.

[32] Diwan V, Chandran SP, Tamhankar AJ, Stalsby Lundborg C, Macaden R (2012). Identification of extended-spectrum beta-lactamase and quinolone resistance genes in Escherichia coli isolated from hospital wastewater from central India. J Antimicrob Chemother.

[33] Diwan V, Tamhankar AJ, Khandal RK, Sen S, Aggarwal M, Marothi Y (2010). Antibiotics and antibiotic-resistant bacteria in waters associated with a hospital in Ujjain, India. BMC Public Health.

[34] Pathak A, Chandran SP, Mahadik K, Macaden R, Lundborg CS (2013). Frequency and factors associated with carriage of multi-drug resistant commensal Escherichia coli among women attending antenatal clinics in central India. BMC Infect Dis.

[35] Pathak A, Mahadik K, Sharma R, Marothi Y, Sharma M, Macaden R (2012). Factors associated with carriage of multi-resistant commensal Escherichia coli among postmenopausal women in Ujjain, India. Scand J Infect Dis.

[36] Shakya P, Barrett P, Diwan V, Marothi Y, Shah H, Chhari N (2013). Antibiotic resistance among Escherichia coli isolates from stool samples of children aged 3 to 14 years from Ujjain, India. BMC Infect Dis.

[37] Nerkar SS, Tamhankar AJ, Khedkar SU, Lundborg CS (2014). Quality of water and antibiotic resistance of Escherichia coli from water sources of hilly tribal villages with and without integrated watershed management-a one year prospective study. Int J Environ Res Public Health.

[38] Sahoo KC, Tamhankar AJ, Sahoo S, Sahu PS, Klintz SR, Lundborg CS (2012). Geographical variation in antibiotic-resistant Escherichia coli isolates from stool, cow-dung and drinking water. Int J Environ Res Public Health.

[39] Sahoo KC, Tamhankar AJ, Johansson E, Stalsby Lundborg C (2014). Community perceptions of infectious diseases, antibiotic use and antibiotic resistance in context of environmental changes: a study in Odisha, India. Health Expect.

[40] Tamhankar AJ, Raghunath D, Nagaraja V, Durgarao C (2009). Location Specific Integrated Antibiotic Resistance Management Strategy (LIARMS). Antimicrobial resistance- the modern epidemic- current status and research issues.

[41] Tamhankar AJ. http://save-antibiotics.blogspot.se/2012/06/antimicrobial-stewardship-issueof.html. Accessed 23 Dec 2015.

